# Alert Village: an awareness and health promotion programme on healthy behaviors

**DOI:** 10.1186/1471-2458-12-S2-A27

**Published:** 2012-11-27

**Authors:** Asmaripa Ainy, Misnaniarti Makky, Nur Alam Fajar

**Affiliations:** 1 Faculty of Public Health, Sriwijaya University, Indralaya, Indonesia

## Background

The spread of non-communicable diseases (NCDs) presents a global crisis and is a major barrier to achievement of the Millennium Development Goals (MDGs). WHO identifies six important risk factors that are associated with NCDs: tobacco use, physical inactivity, overweight/obesity, high blood pressure, high cholesterol levels, and high blood glucose levels. Alert village is an Indonesian government policy implemented since the year 2006, aiming to empower the community to gain healthy living. One of the basic activities in Alert Village is the promotion of healthy behaviors in the community. This study aimed to explore the extent of Alert Village implementation and its role in the promotion of healthy behaviors.

## Materials and methods

A qualitative study was conducted in Sakatiga Village, Ogan Ilir District, Indonesia using three methods: key informant in-depth interview, focus group discussion, and participant-observation. Two health office staffs, a midwife, a village head and two cadres were informants of in-depth interview. Focus group discussion was conducted to six selected community members. Data were analyzed descriptively.

## Results

Results indicated that the extent of alert village implementation had run well. A village forum and a village health post were formed, moreover the midwife and the two cadres were active in dealing with some activities in health post. Some activities for healthy behaviors promotion had already been implemented in the village health post of Sakatiga. Health promotion efforts include: counseling and training of the cadres and the community members namely: promotion of community knowledge regarding NCDs risk factors, healthy food preparation for family and in particular pregnant and lactating mothers and healthy physical activity for elderly. But other facts, community had not actively participated in the implementation of village forum and it worked only when there was a health problem.

## Conclusions

In conclusion, Alert Village program is an acceptable way of health promotion of healthy behaviors in Sakatiga Village. It also has potential in producing beneficial health changes in the community.

